# Forecasting tuberculosis in Ethiopia using deep learning: progress toward sustainable development goal evidence from global burden of disease 1990–2021

**DOI:** 10.1186/s12879-025-11228-3

**Published:** 2025-07-01

**Authors:** Zinabu Bekele Tadese, Fetlework Gubena Arage, Tigist Kifle Tsegaw, Eyob Akalewold Alemu, Tsegasilassie Gebremariam Abate, Eliyas Addisu Taye

**Affiliations:** 1 Department of Health Informatics, College of Medicine and Health Sciences, Samara University, Semera, Ethiopia; 2 Department of Public Health Officer, Institute of Public Health, College of Medicine and Health Sciences, University of Gondar, Gondar, Ethiopia; 3 Department of Epidemiology and Biostatistics, Institute of Public Health, College of Medicine and Health Sciences, University of Gondar, Gondar, Ethiopia; 4 Department of Maternal and Child health, Lemi kura Subcity Health Office, Addis Ababa City Administration Health Bureau, Addis Ababa, Ethiopia; 5 Department of Health Informatics, Institute of Public Health, College of Medicine and Health Sciences, University of Gondar, Gondar, Ethiopia

**Keywords:** Tuberculosis, Machine learning, Deep learning, LSTM, Ethiopia

## Abstract

**Background:**

Tuberculosis (TB) is a preventable and treatable disease caused by *Mycobacterium tuberculosis*, which most often affects lungs and remains the second leading cause of death from infectious diseases worldwide. The National End TB Strategy aims to eliminate the TB epidemic by reducing TB-related deaths by 95% and decreasing incident TB cases by 90% by 2030, using 2015 as the baseline. Tuberculosis is the primary cause of morbidity, ranks third in hospital admissions, and is the second leading cause of death in Ethiopia, following malaria. Hence, this analysis aims to forecast and provide evidence that supports the combined intervention to monitor TB incidence in Ethiopia’s progress toward the Sustainable Development Goals.

**Method:**

Study employed secondary data analysis from the Global Burden of Disease database (1990–2021) to forecast tuberculosis incidence in Ethiopia. LSTM-based models, including multistep LSTM and hybrid ARIMA + LSTM, were implemented for prediction in TensorFlow frameworks while ARIMA model was built using the *statsmodels* and *pmdarima* libraries using the Python programming language. The statistical significance level was set at 0.05 to check data stationarity. Model performance was evaluated using Root Mean Squared Error, Mean Absolute Error, Mean Absolute Percentage Error, and Symmetric Mean Absolute Percentage Error. Finally, the best model was used to forecast the next 9 years from 2021 to 2030.

**Result:**

According to GBD data, the incidence of TB in Ethiopia shows a long-term downward trend, decreasing from 466.93 cases per 100,000 in 1990 to 185.53 by 2021. The analysis result revealed that multistep LSTM model outperformed all achieving MAE: 5.53, RMSE: 6.74, MAPE: 2.72% and sMAPE:2.76%. The incidence of tuberculosis in Ethiopia is projected to decline slightly through 2030, according to a multi-step LSTM model. The forecast estimates that the TB incidence will be 189 cases per 100,000 people by 2025, decreasing further to 179 by 2030.

**Conclusion:**

Overall, the analysis indicates that Ethiopia is still falling short of the national “END TB strategy” goal of 90% reduction in TB incidence cases per 100,000 population by 2030. It highlights the necessity for Ethiopia’s TB control strategies to improve access to prevention, early diagnosis, and treatment, focusing on high-risk groups and vulnerable populations.

**Supplementary Information:**

The online version contains supplementary material available at 10.1186/s12879-025-11228-3.

## Background

Tuberculosis (TB) is a preventable and treatable disease caused by *Mycobacterium tuberculosis* (MTB), that most often affects lungs and yet it remains the second leading cause of death from infectious diseases worldwide [1, 2]. According to a 2023 World Health Organization report, 10.8 million individuals worldwide 6.0 million men, 3.6 million women, and 1.3 million children were predicted to have contracted tuberculosis [3]. The risk of developing this global infectious disease is 18 times higher among HIV-infected individuals [4].

Globally, the annual number of deaths due to tuberculosis decreased from 2010 to 2019; however, this trend reversed in 2020 and 2021 [5]. In the African and South-East Asia regions, it ranked as the fourth and fifth most common cause of death, respectively, and in 2023, tuberculosis likely resumed its position as the top cause of death from a single infectious agent [3, 5]. The projected rise in TB cases from 2021 to 2023 is attributed to interruptions in TB diagnosis and treatment caused by the COVID-19 pandemic, during which the number of newly diagnosed TB patients decreased from 7.1 million in 2019 to 5.8 million in 2020 and 6.4 million in 2021 [6]. However, this is not evident in many countries in Sub-Saharan Africa (SSA), where low case detection caused by missed or delayed diagnoses, drug resistance, and challenges in accessing high-quality care contribute to a higher risk of death, suffering, and severe financial consequences [7].

The National End TB strategy aims to eliminate the TB epidemic by reducing TB-related deaths by 95% and decreasing incident TB cases by 90% by 2030; ensuring that no family faces catastrophic expenses due to TB [8]. The incidence rate was further detailed in the WHO End TB Strategy where it was planned to bring 20% reduction by 2020, 50% by 2025 and 90% by 2030 in the TB incidence rate taking the 2015 incidence (192 per 100,0000 people) as a baseline. To meet the 2030 targets, the country needs to accelerate its annual decline in TB incidence to approximately 19 cases per 100,000 [9, 10]. ​In 2014, the World Health Organization (WHO) introduced a framework eight priority action areas to eliminate TB in low-incidence countries by 2030 [11]. Among key intervention areas, addressing key affected populations, active TB screening and latent TB infection (LTBI) identification in high-risk groups, optimizing multidrug-resistant (MDR)-TB prevention and care, and investing in research and new tools. This framework aims to reach a pre-elimination target for tuberculosis (fewer than 10 cases per million) by 2035 and achieve elimination (fewer than 1 case per million) by 2050 in countries approaching low levels of TB incidence [11, 12]. A targeted and accelerated approach for decreasing TB cases has not yet been implemented or studied in low-income countries with a high TB burden, such as Ethiopia [12].

In Ethiopia, 4 out of 100 people die from TB-HIV co-infection, and the incidence of Multidrug-resistant Tuberculosis (MDR-TB) is estimated at 5.8 per 1,000 people [13]. Reports from Africa identified several factors associated with poor TB treatment success rate such as including old age group [14], HIV infection [15], sputum smear positivity, and previous TB treatment [14]. Poverty may result in poor nutrition, which may be associated with alterations in immune function [16]. It also results in overcrowded living conditions, poor ventilation, and poor hygiene habits which are likely to increase the risk of transmission of TB [17].

In recent decades, the rapid advancements in computer technology have led to a widespread adoption of statistical techniques for modeling and forecasting. Additionally, some studies have utilized deep-learning methods to predict infectious diseases [18–20]. Among the statistical techniques, the autoregressive integrated moving average (ARIMA) method, which relies on the assumption of linearity, is the most widely used for analyzing and assessing the morbidity or mortality time series of infectious diseases, including tuberculosis (TB) [19, 21].

The ARIMA model serves as a technique for examining non-stationary time series data. A key feature of ARIMA analysis is its versatility, as it can be utilized with any time series. Notably, it highlights the intricate changes that occur when the data experiences rapid fluctuations over time [22]. While traditional methods have focused on parametric models informed by domain expertise such as autoregressive (AR) [23] exponential smoothing [24] modern machine learning methods provide a way to learn temporal dynamics in a purely data-driven manner [25].

Traditional forecasting models have poor adaptability to big fluctuation data. Long Short-Term Memory (LSTM) has proven to be a reliable model for forecasting the incidence of diseases [26]. LSTMs were developed because earlier variants of recurrent neural network (RNN) have an infinite lookback window but struggle with learning long-range dependencies in the data [27]. LSTM presents the idea of gates, which include the forget gate, input gate, and output gate. The forget gate controls the retention of information from the previous cell. Two main factors have been recognized as reasons for the underperformance of machine learning methods. First, the inherent flexibility of these methods can lead to overfitting, acting as a double-edged sword [28]. Therefore, simpler models might perform better in situations with limited data, which often occur in forecasting scenarios with few historical observations. Additionally, akin to the stationarity prerequisites of statistical models, machine learning models are also sensitive to the preprocessing of inputs [28, 29].

A recent trend in deep learning involves developing hybrid models that address these limitations, showing improved performance compared to purely statistical or machine learning models across various applications [30, 31]. Hybrid models allow the separation between stationary and non-stationary components, eliminating the necessity for custom input pre-processing [32]. To achieve this objective, ARIMA + LSTM hybrid model was employed, which incorporates the strengths of both linear and nonlinear models to enhance the accuracy of short-term predictions [33]. On the other hand, the multistep LSTM model is a type of recurrent neural network designed for time series forecasting tasks, where predictions are made for multiple future time steps [34]. This analysis aims to forecast and provide evidence that supports the combined intervention to monitor TB incidence in Ethiopia’s progress toward the SDGs by 2030.

## Methods

### Data source and setting

TB data on annual incidence rates from 1990 to 2021 were obtained from the Global Health Disease (GBD) database exchange tool, categorized by sex, year, age, gender, region, and country (https://ghdx.healthdata.org). The database includes the disease burden of 371 diseases or injuries and 88 risk factors worldwide, spanning different geographical regions and 204 countries [35] with subnational level analysis conducted for 21 regions spanning 1990 to 2021. The GBD 2021 project assessed the changes in rates, numbers, and percentages related to incidences, prevalences, deaths, and disability-adjusted life years (DALYs) for various diseases. Detailed estimates of these indices can be found in the appendix of the GBD 2021 capstone paper [36]. The data extraction process is limited to the incidence rate per 100,000 population for the cause of TB in Ethiopia, covering all ages and both sexes from the years 1990 to 2021. Detailed descriptions of analytical methods and models for each cause of disease burden are also available in a searchable online tool https://www.healthdata.org/research-analysis/gbd.

### Case definition

The classification of TB adheres to the International Statistical Classification of Diseases and Related Health Problems (ICD). This includes all forms of TB, both pulmonary and extrapulmonary, whether bacteriologically confirmed or clinically diagnosed. The relevant ICD-10 codes for TB are A10–A14, A15–A19.9, B90–B90.9, K67.3, K93.0, M49.0, N74.1, P37.0, and U84.3 [37].

### Model development

The methodology employed in this analysis integrates both statistical and deep learning models to enhance forecasting accuracy. Preprocessing steps include data normalization using MinMaxScaler from sklearn to ensure stable numerical ranges, which is particularly important for deep learning models. This study used univariate time series data prepared for training a supervised learning model. The data was organized into samples with an input (X) and output (y). For this purpose, the study implemented multiple models, including ARIMA from statsmodels package, LSTM, a hybrid ARIMA + LSTM model, and a multistep LSTM model. Deep learning-based forecasting is implemented using TensorFlow and Keras.

In the ARIMA model, “AR” represents autoregression, describing the past and present data relationship. “I” denotes differential operational. “MA” stands for moving average, which is the sum of the error terms in “AR” [38]. ARIMA model is denoted as ARIMA (*p*, *d*, *q*) where *p* is the order of the autoregressive part, *d* is the order of the differencing, *q* is the order of the moving-average process. To ensure the data meets ARIMA’s stationarity assumption, techniques such as differencing and the Augmented Dickey-Fuller (ADF) test are applied. The test results, including the test statistic, p-value, and critical values, were evaluated to assess stationarity. If the p-value is below the significance level (typically 0.05), the null hypothesis of a unit root is rejected, indicating a stationary time series. These analyses were crucial, as they allowed us to determine the appropriate values for p and q. With the stationarity of the data confirmed, an ARIMA model was developed. Parameters were selected based on the autocorrelation function (ACF) and partial autocorrelation function (PACF) plots, which help identify the appropriate values for p and q. Additionally, a rolling mean generated to observe changes in statistical properties over time as (Eq. 1) where *xi*: the value of the time series at time point *i*,* t*: current point time, *w*: window size number of consecutive data points considered for each calculation and $\:\stackrel{-}{{x}_{t}}$: the rolling mean at time *t.*

1 $$\overline {{x_t}} = \frac{1}{w}\sum\nolimits_{i = t - w + 1}^t {{x_i}} $$

Long-short term memory (LSTM) is a machine learning algorithm with recurrent neural network architecture [39]. As a model, it stores the information learned in the short period and uses it for training in the long period. Therefore, long short-term memory contains units called “memory blocks” in hidden layer. These memory blocks can be defined as hidden units in traditional repeating neural networks. It contains one or more memory cells in the memory blocks. Each memory block contains input and output ports to control the flow of information. While the input gate controls the flow of input activation information in the memory cell, the output doors control the flow of output activation information. Later, a “forget gate” was added to the memory blocks. The forgetting gate scales the internal state of the cell, resets the memory of the cell, before the input activation through the cell’s repetitive connection [40].

LSTM neural network was developed with two hidden layers of 50 units each, both using ReLU activation function. we utilized a sliding window approach with a sequence length of 4 (i.e., each input sample included 4 past time steps to predict the next value). The dataset was split using a 10-fold TimeSeriesSplit cross-validation method to maintain temporal order and avoid look-ahead bias. In each fold, the LSTM model was trained on the training set and evaluated on the validation set. Models used the Adam optimizer (learning rate: 0.001), trained for up to 300 epochs with early stopping (patience: 10) based on training loss, and a batch size of 32. After retraining on the full dataset, a 9-step ahead forecast was generated iteratively using the last 4 time steps as input, with outputs inverse-transformed to the original scale.

To further enhance forecasting performance, a multistep LSTM model is implemented. Unlike single-step LSTM models that predict only the next time step, the multistep model forecasts multiple future time steps simultaneously. The architecture involves defining an appropriate input-output window size, where past observations serve as inputs to predict multiple future points. This model directly predicts the next 9-time steps in a single forward pass using the same input sequence of 4-time steps. The architecture similarly consisted of two LSTM layers with 50 units each, followed by a dense output layer with 9 neurons. Training used the Adam optimizer (learning rate = 0.001), with up to 100 epochs per fold and early stopping (patience = 10) during 10-fold cross-validation, followed by 300 epochs of training on the full dataset. Final forecasting was conducted using the last 4 time points to output a 9-year ahead forecast, which was then inverse-scaled to return to the original value range.

Additionally, a hybrid ARIMA + LSTM model is developed to leverage the strengths of both approaches. ARIMA is first applied to capture linear components of the time series, and the residual errors (representing nonlinear patterns) are modeled using an LSTM network. This hybrid approach aims to improve prediction accuracy by combining the statistical power of ARIMA with the feature extraction capability of LSTMs.

### Model selection and feature forecasting

Performance evaluation is conducted using standard error metrics, including Root Mean Square Error (RMSE), Mean Absolute Error (MAE), Mean Absolute Percentage Error (MAPE), and symmetric Mean Absolute Percentage Error (sMAPE) calculated as (Eq. 2 to 5) where, $\:\widehat{{y}_{i}}$ is the predicted value, $\:{y}_{i}$ is the observed value, and *n* is the series length.

2 $$\:RMSE = \sqrt {\frac{1}{n}\sum\nolimits_{i = 1}^n {{{\left( {\widehat {{y_i}} - {y_i}} \right)}^2}} } $$

3 $$\:MAE = \frac{1}{n}\sum\nolimits_{i = 1}^n {\left| {\widehat {{y_i}} - {y_i}} \right|} $$

4 $$\:MAPE = \frac{{100\backslash \:\% }}{n}\sum\nolimits_{i = 1}^n {\left| {\frac{{\widehat {{y_i}} - {y_i}}}{{{y_i}}}} \right|} $$

5 $$\:sMAPE = \frac{{100\backslash \:\% }}{n}\sum\nolimits_{i = 1}^n {\frac{{2\left| {\widehat {{y_i}} - {y_i}} \right|}}{{\left| {\widehat {{y_i}}} \right| + \left| {{y_i}} \right|}}} $$

## Results

**Fig. 1:**
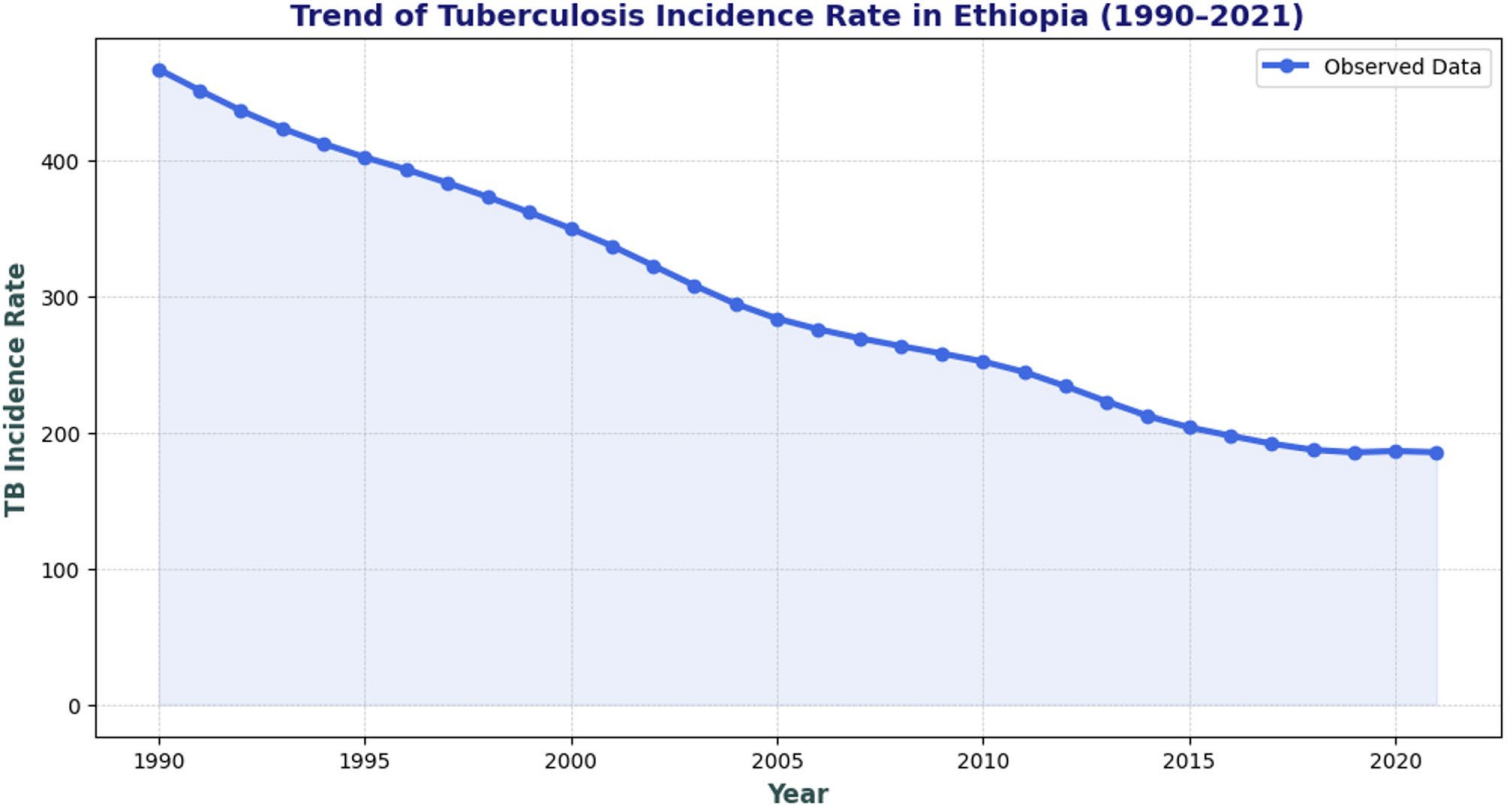
Tuberculosis incidence in Ethiopia 1990–2021

According to GBD data, the incidence of TB in Ethiopia shows a long-term downward trend, decreasing from 466.93 cases per 100,000 in 1990 to 185.53 by 2021 (Fig. 1).

### ARIMA

**Fig. 2:**
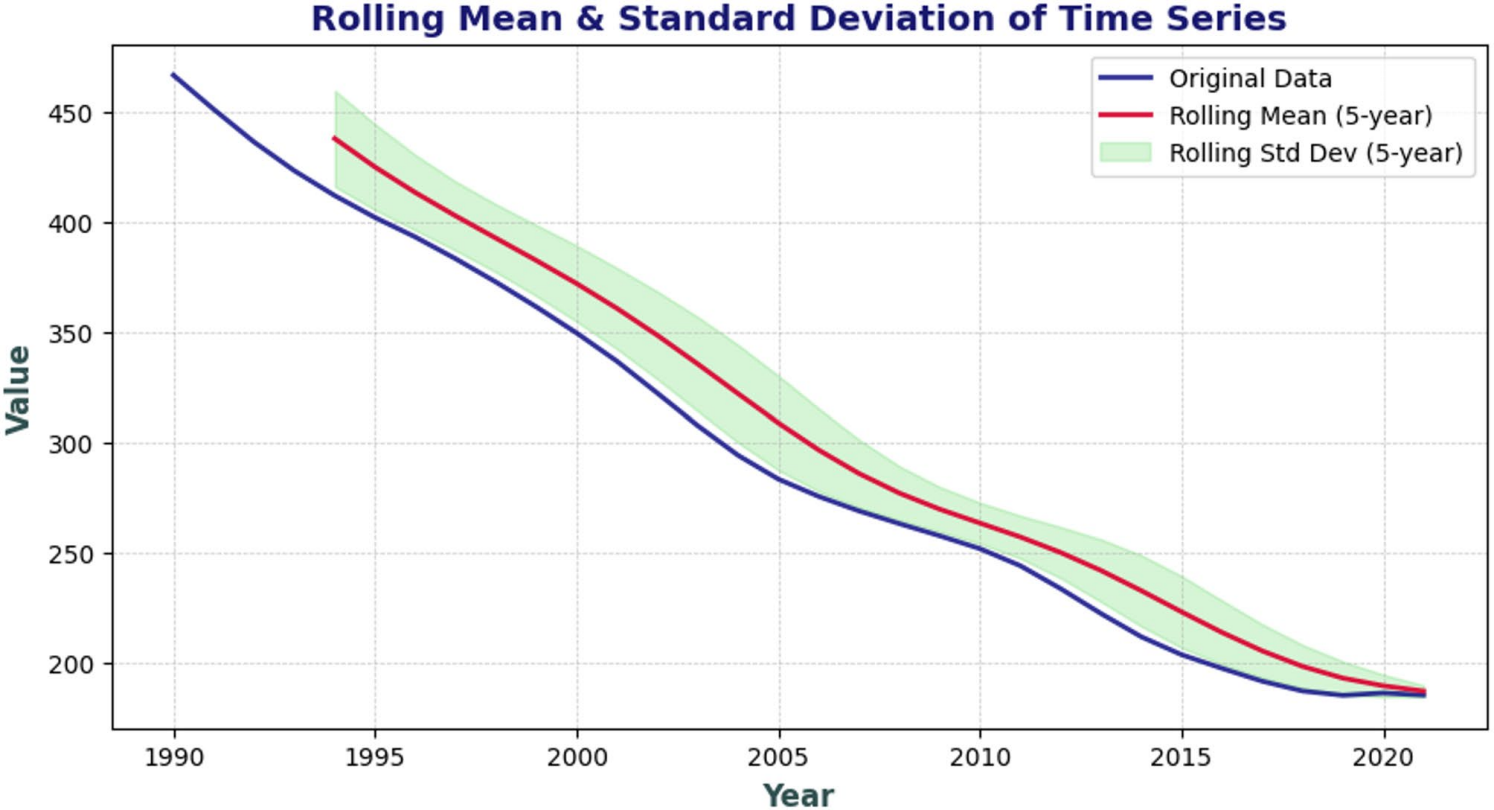
Stationary check using rolling mean and rolling standard deviation

To effectively analyze the data using an ARIMA model, we began by testing for stationarity over time, which is a critical step in the process. The rolling mean (Fig. 2) closely follows the original data, indicating a changing mean over time and reinforcing the non-stationary nature of the series. A stationary time series would exhibit a relatively stable mean, which is absent here. Additionally, the rolling standard deviation does not exhibit large variations, suggesting that while the data trends downward, its overall variability is relatively stable. Recognizing the possibility of underlying periodic patterns, we conducted a seasonality check through decomposition analysis, which revealed that there is no seasonality in our data (Supp1).

**Fig. 3:**
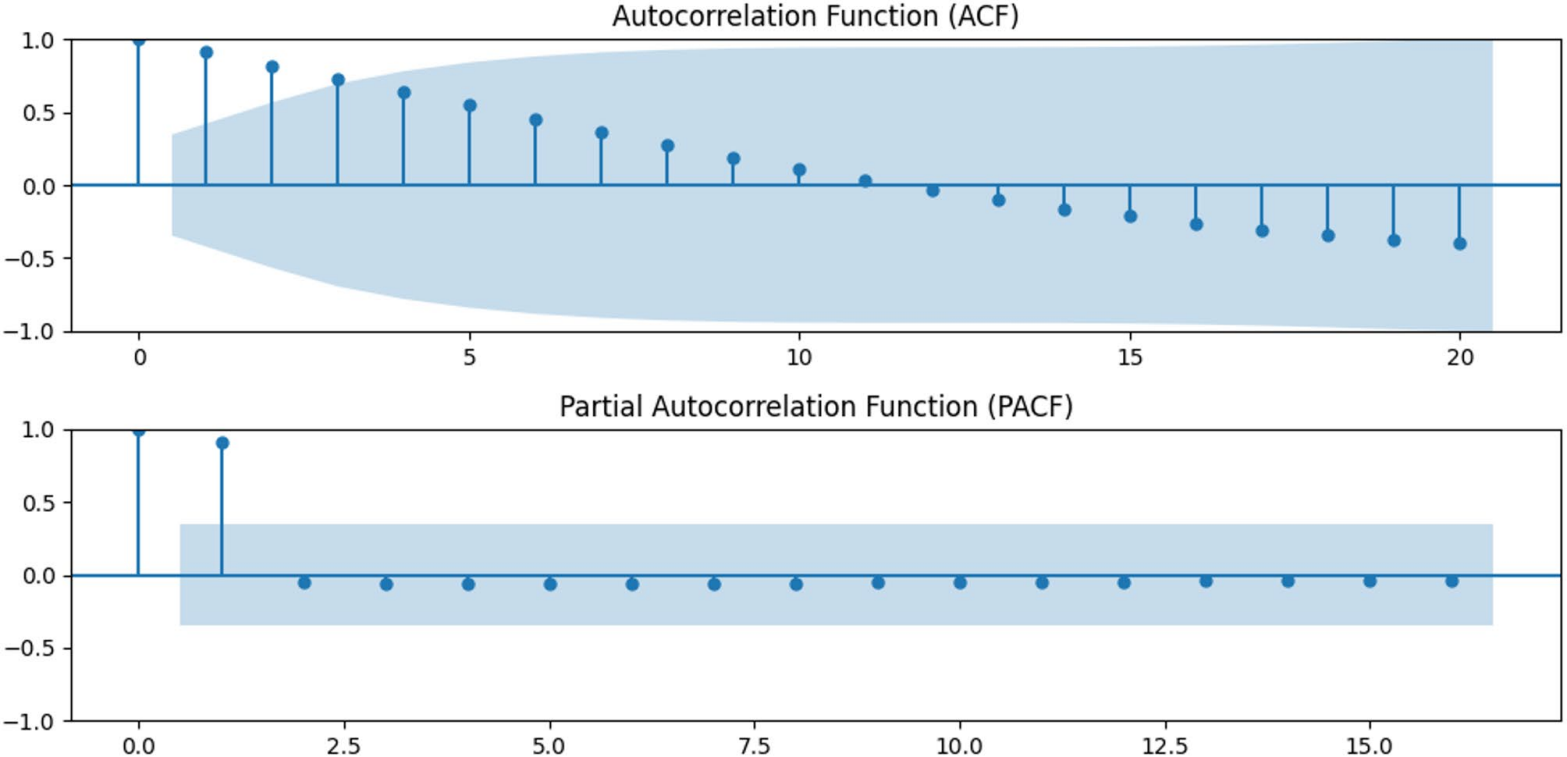
Autocorrelation function and partial autocorrelation function

**Fig. 4:**
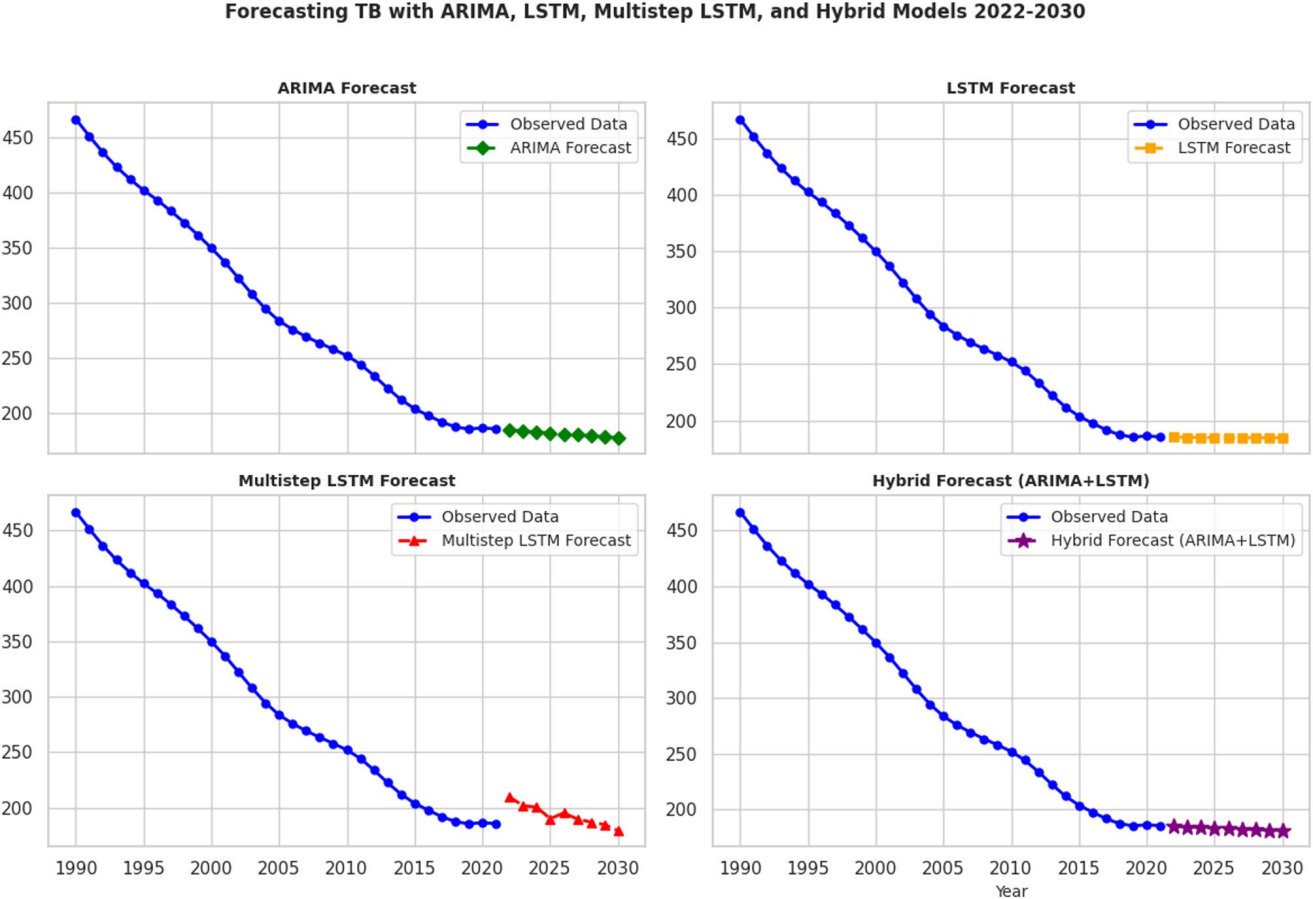
Prediction of TB incidence in Ethiopia, 2022–2030

Given the observed trend and the rolling mean and rolling standard deviation indicating potential non-stationarity, it’s essential to perform the ADF test to verify non-stationarity. In this case, the ADF statistic of -1.51 and p-value of 0.52 provide strong evidence in favor of the null hypothesis, confirming that the time series is non**-**stationary. Given the non-stationarity of our time series, we needed to make it stationary through differencing. To further refine our model selection, we analyzed the autocorrelation (ACF) and partial autocorrelation (PACF) graphs (Fig. 3). Ultimately, the best order identified for our model was ARIMA (1,1,0). The model is then fitted to the data and forecasted to the next 9 years (Fig. 4), (Table 1). Additional information is available from the supplementary file (supp1).

### LSTM, multistep LSTM, hybrid or ARIMA + LSTM

In addition to the ARIMA model, other models such as LSTM (Long Short-Term Memory) are also developed and evaluated. LSTM is a type of recurrent neural network (RNN) that is particularly well-suited for time series forecasting due to its ability to capture long-term dependencies in the data [39]. The development process began with a traditional LSTM model for single-step forecasting. The raw time series data was first scaled using MinMaxScaler, then restructured into sequences of four-time steps, with each sequence paired with its subsequent target value. A 10-fold TimeSeriesSplit cross-validation approach was employed to assess model performance, using an Adam optimizer (with a learning rate of 0.001) and an EarlyStopping callback to prevent overfitting. For instance, mean squared error (MSE) of each 10 folds for multistep LSTM model was 0.006, 0.003, 0.007, 0.008, 0.005, 0.007, 0.007, 0.010, 0.007, 0.004, 0.006, 0.005, 0.004, 0.006, 0.007, 0.005, 0.008, 0.020, 0.011, 0.002. The final LSTM model was retrained on the full dataset and used iteratively to forecast multiple future points by feeding its predictions back as inputs (Fig. 4). Additional information is available from the supplementary file (supp1).

In contrast, the multistep LSTM approach was designed to forecast nine steps ahead in a single pass. Here, the input sequences were constructed similarly with a length of four, but each sequence’s target output comprised the next nine values. This model, also built with two LSTM layers followed by a Dense layer outputting nine values, was evaluated via cross-validation and then retrained on the entire dataset with the same early stopping mechanism. Forecasts generated by this model were directly transformed back to the original scale (Fig. 4), (Table 1). Additional information is available from the supplementary file (supp1).

Finally, to leverage the strengths of both statistical and deep learning methods, a hybrid model was created by averaging the forecasts from an ARIMA model (which excels in capturing linear trends and seasonality) and the LSTM model (which effectively captures complex nonlinear patterns). This combined ARIMA + LSTM approach aims to produce more robust predictions by balancing the strengths and compensating for the limitations of each method. Unfortunately, the hybrid model did not perform as well as the other model, with an MAE of 13.97, RMSE of 18.05, MAPE of 6.77, and sMAPE of 7.15.

### Model comparison and final predictions

After developing final models based on the optimal hyperparameters on the training data, the performance of the fitted models on the test data was assessed as shown in Tables 1and Supp1. The results showed that multistep LSTM model was the best with MAE: 5.53, RMSE: 6.74, MAPE: 2.72 and sMAPE:2.76. This means MAE suggests that, on average, predictions deviate by 5.53 units from actual values. Additionally, MAPE2 and sMAPE indicated that the model’s predictions have a very low relative error, confirming its high accuracy. The least performing among the four models was ARIMA with MAE:15.97, RMSE: 19.09, MAPE: 7.82 and sMAPE:8.25.

**Table 1:** Model comparison and performance evaluation result

Models	MAE	RMSE	MAPE	sMAPE
ARIMA	15.97	19.10	7.82	8.26
LSTM	11.98	17.24	5.72	6.07
Multistep LSTM	5.54	6.75	2.72	2.77
ARIMA + LSTM	13.98	18.05	6.77	7.16

The incidence of tuberculosis (TB) in Ethiopia is projected to decline slightly through 2030, according to a multi-step LSTM model (see Table 2; Fig. 4). The forecast estimates that the TB incidence will be 189 cases per 100,000 people by 2025, decreasing further to 179 by 2030. However, this projection indicates that Ethiopia is still falling short of the WHO “END TB strategy” [9] goal of a 80% reduction in TB incidence cases per 100,000 population by 2030.

**Table 2:** Forecasting of TB incidence in ethiopia, 2021–2030

Year	ARIMA	LSTM	Multistep LSTM	Hybrid ARIMA + LSTM
2022	184.60	185.36	209.48	184.98
2023	183.67	185.13	201.75	184.40
2024	182.76	185.15	200.38	183.95
2025	181.85	184.98	189.98	183.42
2026	180.96	184.93	195.16	182.95
2027	180.08	184.89	189.74	182.48
2028	179.20	184.86	186.93	182.03
2029	178.34	184.83	184.68	181.59
2030	177.49	184.82	179.25	181.15

## Discussion

In the present study the incidence of TB in Ethiopia shows a long-term downward trend, decreasing from 466.93 cases per 100,000 in 1990 to 185.53 by 2021 from data obtained from GBD. This study utilized a variety of comprehensive time series methods, which included statistical tests such as ARIMA, deep learning techniques like LSTM and multistep LSTM, as well as a hybrid approach combining ARIMA and LSTM, to predict tuberculosis incidence in Ethiopia. Model performance was evaluated using multiple metrics including MAE, RMSE, MAPE, and sMAPE, which collectively demonstrated the model’s predictive capability. The analysis result revealed that multistep LSTM model outperformed all achieving MAE: 5.53, RMSE: 6.74, MAPE: 2.72% and sMAPE: 2.76%. Reports indicate that RNNs with memory, like LSTM, outperform ARIMA by a significant margin, while models that process data in both forward and backward directions provide better prediction accuracy compared to unidirectional LSTM models [41]. In our finding ARIMA was the lowest performed model from all. This finding is consistent with [42], which highlighted that ARIMA performs poorly when dealing with complex time series data when compared to LSTM, multistep LSTM and hybrid model. The goals outlined in the End TB Strategy aim for an incidence of 2.4 tuberculosis cases per 100,000 people and 444 TB-related deaths in the EU/EEA by 2030 [43]. Ethiopia is among the 30 High TB Burden Countries, with an annual estimated TB incidence of 177/100,000 population and a death rate of 25 per 100,000 population in 2016 [44]. The aim of SDG target 3.3 is to eliminate the epidemics of AIDS, tuberculosis, malaria, and neglected tropical diseases, as well as to tackle hepatitis, waterborne illnesses, and other communicable diseases by the year 2030. The WHO End TB Strategy outlined specific targets to reduce the incidence of tuberculosis (both new and relapse cases) by 20% by 2020, 50% by 2025, and 90% by 2030, using the incidence rate from 2015 as a baseline. Additionally, it aimed for a 35% reduction in annual TB deaths by 2020, 75% by 2025, and 90% by 2030 [9]. Ethiopia is among these seven countries which already achieved the 2020 WHO End TB strategy milestone. The three pillars of End TB strategy are integrated patient-centered care and prevention, bold policies and support systems and intensified researches and innovations.

Our analysis forecasts that by 2025, the TB incidence will reach 189 cases per 100,000 population and by 2030, it will drop to 179 cases, which does not meet the goal. Another study used data from GBD and forecasted the incidence of TB in the Tigray region of Ethiopia to reach 372 by 2025 [45]. This may be due to the health system’s issues since 2019, including COVID-19 and political instabilities, which have hindered access to treatment. A research study involving countries from the USAID TB portfolio highlights how COVID-19 has hindered the global TB response: not only by leading to increases in global TB burden but also complicating the monitoring of the progress toward TB elimination [46]. Articles indicate that diversion of resources towards COVID-19 research and development may contribute to the slowdown in TB research and the development of new vaccines, highlighted by the increasing funding gap for TB research [47]. Limited access to healthcare services in low-income countries delay diagnosis and treatment which lead to contributing factor in the spread of TB and over 80% of TB cases and deaths [48]. To effectively reduce TB incidence in Ethiopia, it is essential to implement annual mass screening targeted at subpopulations with limited access to healthcare, particularly in impoverished and remote regions [12]. Moreover, immediate optimization of tuberculosis control through the utilization of existing technology, along with the simultaneous advancement of a more powerful diagnostics, pharmaceuticals, and vaccines is essential [49].

### Strengths and limitations

This study assessed the incidence of TB within the population using data from the GBD, which is considered both authoritative and trustworthy. The forecasting model clearly reveals the epidemic pattern of TB through assumptions, parameter estimation, and fitting validation, providing a theoretical foundation for developing prevention strategies and interventions. Furthermore, this research significantly contributes to TB prevention literature within the context of SDG goals, particularly regarding deep learning methodologies. Our research had a few limitations. First, the GBD data was updated until 2021, and more recent figures from 2022 were not yet accessible to confirm the findings of this study. Second, the incidence predictions were assessed solely through time series analysis; incorporating additional variables might have yielded more dependable outcomes. Also, the case definition used as outcome of this study was based on ICD codes and taken from previous published study. Moreover, no prior research has employed deep learning techniques in Ethiopia to benchmark the results against actual data alongside our findings.

## Conclusion

This analysis utilized ARIMA, LSTM, multistep LSTM, and a hybrid ARIMA + LSTM model to forecast tuberculosis (TB) incidence in Ethiopia. Among these methods, the multistep LSTM model showed the best performance. Although Ethiopia met the World Health Organization (WHO) milestone for 2020, increased efforts are necessary to achieve the 2030 goal. The result underscores the importance of Ethiopia’s TB control strategies in enhancing access to prevention, early diagnosis, and treatment, with a focus on high-risk groups and vulnerable individuals. In addition, increased funding, appropriate monitoring, strengthening of health systems, and enhancing national and regional surveillance for TB disease are crucial.

## Electronic supplementary material

Below is the link to the electronic supplementary material.

Supplementary Material 1

## Data Availability

The data sets generated during and or analyzed for the study are available in the IHME data repository and can be accessed directly from https://vizhub.healthdata.org/gbd-results/. with terms and condition clearly stated as “Data made available for download on IHME Websites can be used, shared, modified or built upon by non-commercial users in accordance with the IHME free-of-charge non-commercial user agreement”.

## Abbreviations

ARIMA: Autoregressive integrated moving average
ADF: Augmented Dickey-Fuller
GBD: Global burden of disease
LSTM: Long-short term memory
MAE: Mean absolute error
MAPE: Mean absolute percentage error
sMAPE: symmetric mean absolute percentage error
PACF: Partial autocorrelation function
RMSE: Root mean squared error
RNN: Recurrent neural network
