# Supplementary material for: Forecasting tuberculosis in Ethiopia using deep learning: progress toward sustainable development goal evidence from global burden of disease 1990–2021
Source: BMC Infect Dis. 2025 Jul 1;25:870. doi: 10.1186/s12879-025-11228-3 (PMC12218079; doi:10.1186/s12879-025-11228-3)
Supplement: Supplementary file 1 — Supplementary Material 1 [file 12879_2025_11228_MOESM1_ESM.docx]

**Forecasting Tuberculosis in Ethiopia Using Deep Learning: Progress Toward Sustainable Development Goal Evidence from Global Burden of Disease**

**Supplementary file1**


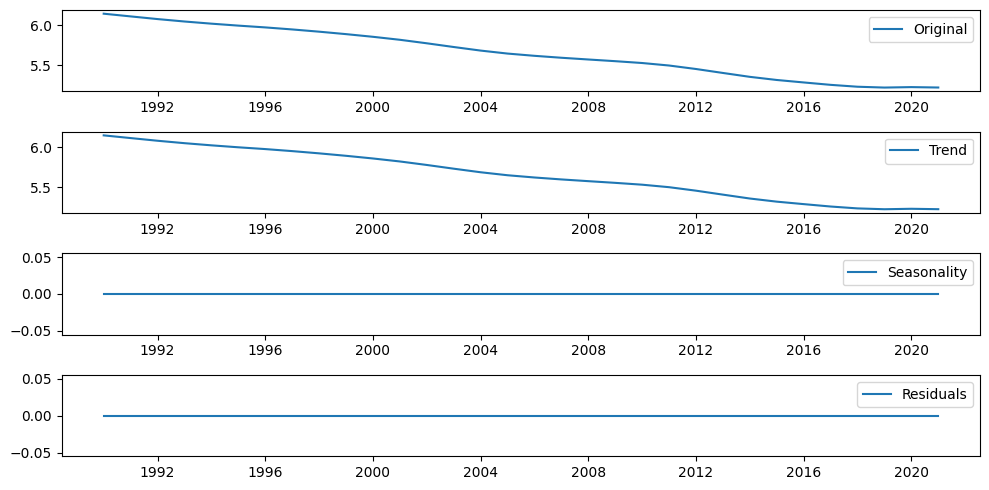


Trend, residual and Seasonality check


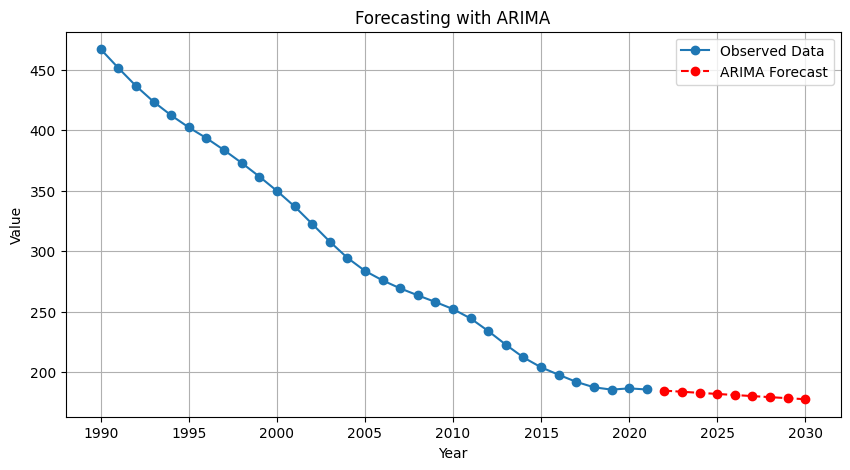

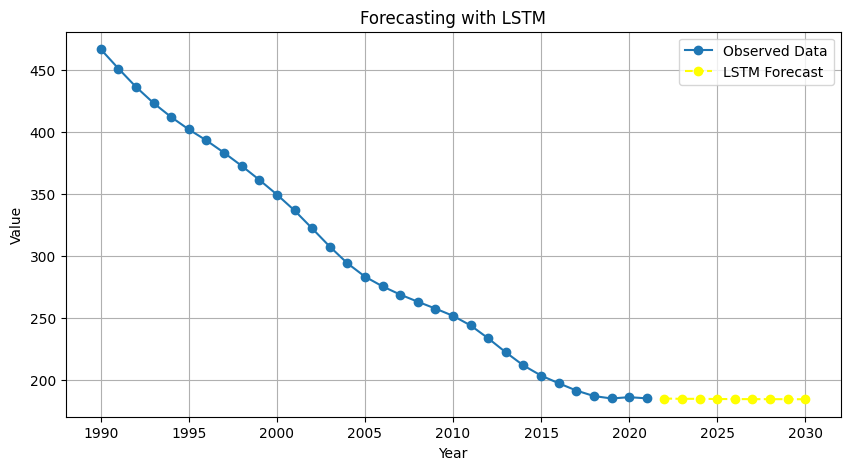

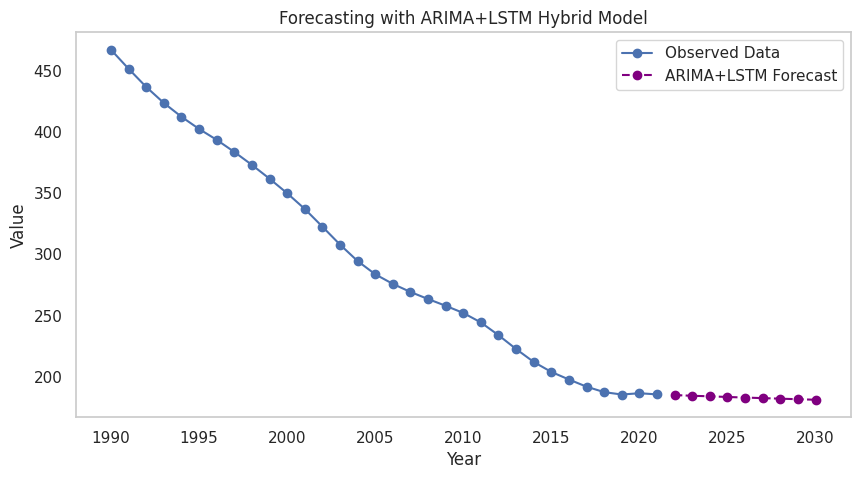

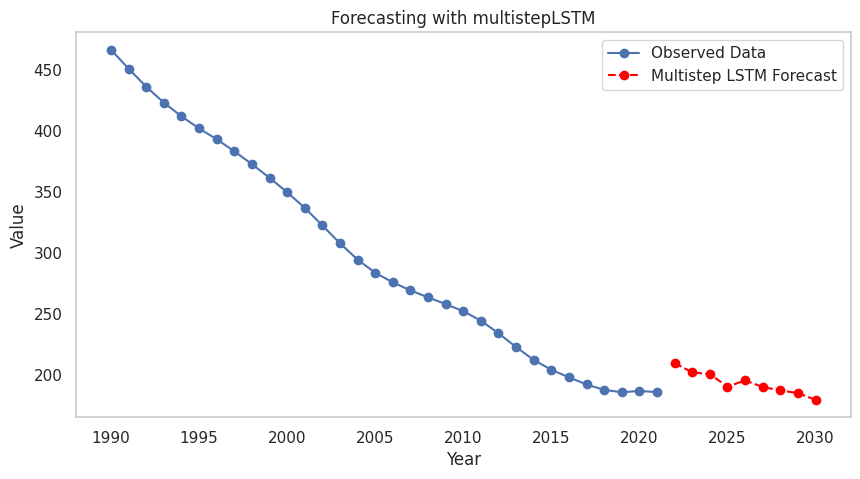

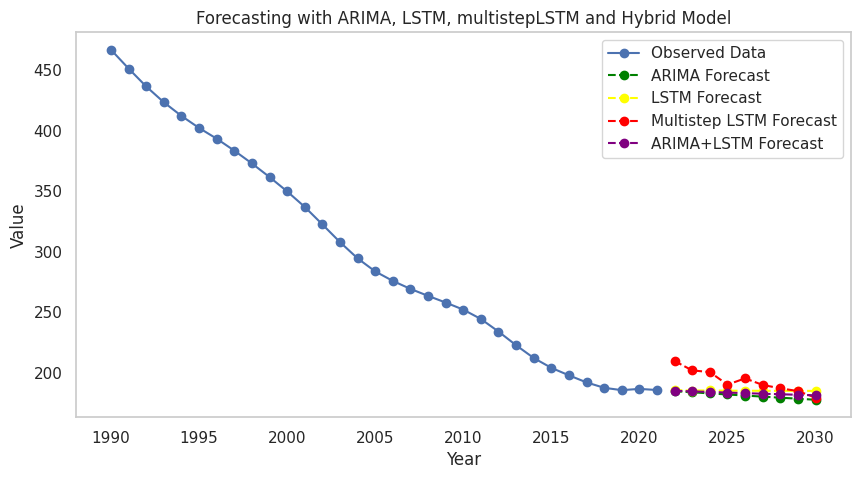


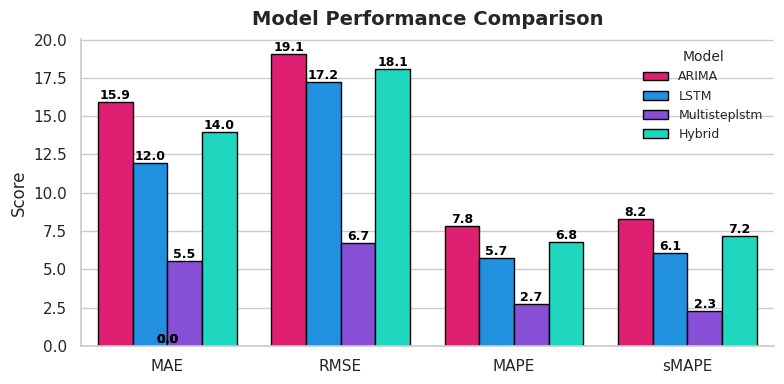


Model comparison and performance evaluation result
